# The crucial role of blood VEGF kinetics in patients with locally advanced esophageal squamous cell carcinoma receiving curative concurrent chemoradiotherapy

**DOI:** 10.1186/s12885-018-4731-9

**Published:** 2018-08-20

**Authors:** Yen-Hao Chen, Hung-I Lu, Chien-Ming Lo, Yu-Ming Wang, Shang-Yu Chou, Chang-Chun Hsiao, Chao-Cheng Huang, Li-Hsueh Shih, Su-Wei Chen, Shau-Hsuan Li

**Affiliations:** 1 Department of Hematology-Oncology, Kaohsiung Chang Gung Memorial Hospital and Chang Gung University College of Medicine, Kaohsiung, Taiwan; 2 Graduate Institute of Clinical Medical Sciences, College of Medicine, Chang Gung University, Taoyuan, Taiwan; 3 School of Medicine, Chung Shan Medical University, Taichung, Taiwan; 4 Department of Thoracic and Cardiovascular Surgery, Kaohsiung Chang Gung Memorial Hospital and Chang Gung University College of Medicine, Kaohsiung, Taiwan; 5 Department of Radiation Oncology, Kaohsiung Chang Gung Memorial Hospital and Chang Gung University College of Medicine, Kaohsiung, Taiwan; 6 Center for Shockwave Medicine and Tissue Engineering, Kaohsiung Chang Gung Memorial Hospital, Kaohsiung, Taiwan; 7 Department of Pathology, Kaohsiung Chang Gung Memorial Hospital and Chang Gung University College of Medicine, Kaohsiung, Taiwan; 8 Biobank and Tissue Bank, Kaohsiung Chang Gung Memorial Hospital, Kaohsiung, Taiwan; 9 Department of Nursing, Kaohsiung Chang Gung Memorial Hospital, Kaohsiung, Taiwan; 10 Department of Anesthesia, Kaohsiung Medical University Hospital, Kaohsiung, Taiwan

**Keywords:** VEGF, Esophageal cancer, Squamous cell carcinoma, Concurrent chemoradiotherapy

## Abstract

**Background:**

To evaluate the role of blood vascular endothelial growth factor (VEGF) kinetics in patients with locally advanced esophageal squamous cell carcinoma (ESCC) receiving curative concurrent chemoradiotherapy (CCRT).

**Methods:**

A total of 97 locally advanced ESCC patients were enrolled. All the patients had their blood drawn at three time points to determine their levels of VEGF, including pre-chemotherapy (day 0), post-chemotherapy (day 5), and pre-cycle 2 chemotherapy (day 28). The VEGF levels were evaluated according to the day 0 value, day 5 value, day 28 value, day 5/day 0 ratio, day 28/day 0 ratio, and day 28/day 5 ratio. A VEGF cut-off level of 80 pg/mL was applied.

**Results:**

In the analysis of progression-free survival (PFS), the patients less than 60 years old had significantly superior PFS compared to those more than 60 years old. Patients who had VEGF < 80 pg/mL at day 28 and a day 28/day 5 ratio < 1 had better PFS than those with VEGF > 80 pg/mL and a day 28/day 5 ratio > 1, respectively. In the analysis of overall survival (OS), patients with N0–1 status had significantly superior OS compared to those with N2–3 status. Furthermore, patients who had VEGF < 80 pg/mL at day 28, a day 5/day 0 ratio < 1, and a day 28/day 5 ratio < 1 had superior OS compared to those patients with VEGF > 80 pg/mL, a day 5/day 0 ratio > 1, and a day 28/day 5 ratio > 1, respectively. In the multivariate analysis, only VEGF < 80 pg/mL at day 28 and a day 28/day 5 ratio < 1 represented independent prognostic factors of superior PFS and OS.

**Conclusions:**

Our study suggests that VEGF kinetics is a prognostic factor for locally advanced ESCC patients receiving curative CCRT. For these patients, lower post-treatment VEGF levels and decreasing levels of VEGF during CCRT are significantly associated with better clinical outcomes.

## Background

Esophageal squamous cell carcinoma (ESCC) is one of the most fatal human malignancies with an increasing incidence worldwide. In Taiwan, ESCC ranks the ninth leading cause of cancer-related deaths [1]. At present, surgery is the main treatment for early stage ESCC; however, most ESCC patients were diagnosed with initial presentation of dysphagia, contributing to locally advanced disease and clinical unresectable status. For these locally advanced ESCC patients, concurrent chemoradiotherapy (CCRT) is one of the standard treatment modalities rather than radical esophagectomy. However, even though there were significant development in surgical technique, medical treatment and supportive care, the clinical prognosis of these ESCC patients remains unsatisfactory [2–6]. Local recurrence, metastatic spread, and resistance to chemotherapy/radiotherapy contribute to the poor prognosis of these patients. Therefore, identifying a robust predictive biomarker or biomarkers to guide therapeutic selection is very important.

Angiogenesis implies complex cellular and molecular interactions between tumor cells, soluble chemokines/cytokines and extra-cellular matrix components, and plays a crucial role in the tumor cell proliferation, invasion, migration, metastasis and disease progression. Folkman et al. reported that the development of new blood vessels are necessary to feed tumor cells themselves, and promote the proliferation, migration and metastasis of tumor cells; furthermore, several previous historical observations have demonstrated the crucial role of angiogenesis in cancer development [7]. The molecular factors of angiogenesis have been characterized by previous studies, and vascular endothelial growth factor (VEGF) family is the most prominent stimulating angiogenic factor, contributing to the foremost controller of physiological and pathological angiogenesis. VEGF can be produced by tumor cells, macrophages, platelets, stroma, and other host cells, and plays a significant role in various aspects of cancer development, such as tumor cell differentiation, the promotion of tumor cell invasion and migration, endothelial cell proliferation, and increasing the vascular permeability of endothelial cells [8–11]. Several studies have revealed that elevated baseline levels of VEGF are related to worse outcomes following chemoradiotherapy in esophageal cancer patients [12, 13]. Moreover, growing evidences have shown that the blocking of VEGF-mediated signaling pathways can potentiate the effect of radiotherapy [14]. However, the correlation, if any, between clinical outcomes and changes in VEGF levels over the course of CCRT in ESCC patients remains unclear.

In the present study, locally advanced ESCC patients who underwent CCRT as a curative treatment in our hospital were enrolled. Blood samples were collected from the patients before and after chemotherapy in order to determine their serum levels of VEGF. The aim of the study was to evaluate the role of VEGF levels in locally advanced ESCC patients receiving curative CCRT.

## Methods

### Patient selection

Any patients with locally advanced ESCC who were treated with curative CCRT at Kaohsiung Chang Gung Memorial Hospital between January 2010 and December 2015 were considered potential candidates for this study. Of those patients, however, any with a history of second primary malignancy and any who had distant metastasis were excluded. We also excluded any patients who received any other therapeutic modality, such as surgical resection or induction chemotherapy, before receiving curative CCRT. Finally, a total of 97 ESCC patients were enrolled. These patients had their blood drawn at the following three time points to determine their levels of VEGF at those time points: pre-chemotherapy (day 0), post-chemotherapy (day 5), and pre-cycle 2 chemotherapy (day 28).

Each ESCC patient included in the study underwent chest computed tomography (CT), endoscopic ultrasonography (EUS), and positron emission tomography (PET) scans to determine the clinical tumor stage according to the 7th American Joint Committee on Cancer staging system. These tests were performed according to the protocol described in the previously published studies [15, 16].

### Serum VEGF measurement

Peripheral blood samples were drawn into sterile glass tubes after an overnight fast, allowed to coagulate at room temperature for 30 min, and then centrifuged at 2000 g for 10 min. After that, the serum was separated, aliquoted, and stored at − 70 °C until assay. The serum samples of the 97 ESCC patients were measured for circulating VEGF using a commercially available enzyme-linked immunosorbent assay (ELISA) kit (Quantikine; R&D Systems Abingdon, UK). The manufacturer’s protocols were followed, and the samples were measured in duplicate, with the mean value being taken as the final concentration. ELISA plates were read using the Emax Precision Microplate Reader (Molecular Devices, Sunnyvale, CA, USA). Standard curves were generated and sample values were determined. Intra-assay and inter-assay validation were also performed. Subsequently, a total of 6 VEGF values were determined for each patient, including a day 0 value, day 5 value, day 28 value, day 5/day 0 ratio, day 28/day 0 ratio, and day 28/day 5 ratio. The normal range of VEGF for healthy subjects in East Asia was found to be 79.6 ± 39.2 pg/mL in a previous study, so the value of 80 pg/mL was selected as the cut-off level in this study [17].

### CCRT planning

The details of the radiotherapy (RT) treatment were as follows. All of the patients underwent CT-simulation and were immobilized in customized thermoplastic devices. The images were acquired from the upper neck through the upper pelvis with 5 mm slice thickness. The patients’ target volumes were then delineated by their treating radiation oncologists. The gross target volume (GTV) covered all of the gross tumor and LNs noted on pre-treatment CT and PET-CT images. The clinical target volume (CTV) encompassed the entire esophagus and mediastinum to cover all LN routes. The planning target volume (PTV) was generated from the corresponding CTV with 1.0–2.0 cm expansion in all directions. The three-dimensional conformal radiotherapy (3D-CRT) technique or the intensity-modulated radiotherapy (IMRT) technique with 6-MV or 10-MV photon beams were used for treatment planning and radiation delivery. The prescribed dose to the PTV was 50–50.4 Gy in 25–28 daily fractions, 5 days a week.

Chemotherapy consisted of cisplatin (75 mg/m^2^; 4-h infusion) on day 1 and 5-fluorouracil (1000 mg/m^2^; continuous infusion) on days 1–4 every 4 weeks, and was arranged concurrently with radiotherapy. For patients with creatinine clearance < 60 mL/min, carboplatin was used instead of cisplatin. The chemotherapy and radiotherapy were administered according to the protocol described in the previously published studies [15, 16].

### Statistical analysis

Comparisons between the groups were performed using the chi-square test, Fisher’s exact test, and *t*-test for categorical variable data. Progression-free survival (PFS) was calculated from the date of starting treatment for the esophageal cancer to the date of disease progression or death from any cause, and overall survival (OS) was calculated from the date of diagnosis of the esophageal cancer to the date of death or last contact. The Kaplan–Meier method was used to estimate PFS and OS, and the log rank test was performed to evaluate the differences between the groups for univariate analysis. Multivariate analyses of the prognostic factors for survival were performed using the Cox proportional hazards model. The hazard ratios with 95% confidence intervals and *P* values were calculated to quantify the strength of the associations between the prognostic parameters and survival. The statistical analyses were performed with the SPSS 19 software package (IBM, Armonk, NY). All of the tests were two-sided tests, and *P* < 0.05 was considered statistically significant.

## Results

### Patient characteristics

Locally advanced ESCC patients who received curative CCRT in our hospital were selected. These patients all had their blood drawn in order to measure their levels of VEGF at three time points, including the pre-chemotherapy (day 0), post-chemotherapy (day 5), and pre-cycle 2 chemotherapy (day 28) time points. Of the 97 ESCC patients who were ultimately enrolled, 96 were men and only one was a woman, and they had a mean age of 56 years (range: 32 to 77 years). The tumor T status was found to be T1 in four (4%) patients, T2 in 11 (11%) patients, T3 in 29 (30%) patients, and T4 in 53 (55%) patients. Meanwhile, four (4%) patients were diagnosed as having N0 status, 27 (28%) patients were diagnosed as having N1 status, 39 (40%) patients were diagnosed as having N2 status, and 27 (28%) patients were diagnosed as having N3 status. In terms of tumor stage according to the AJCC 7th staging system indicated that four (4%) patients had stage II, 13 (13%) patients had stage IIIA, 14 (15%) patients had stage IIIB, and 66 (68%) patients had stage IIIC. Among the 97 patients, 23 (24%) patients were classified as grade 1, 48 (49%) patients as grade 2, and 26 (27%) patients as grade 3. The primary tumor location showed that 40 (41%) patients had tumor located in the upper esophagus, 34 (35%) patients in the middle esophagus, and 23 (24%) patients in the lower esophagus. Among the 97 patients, 88 (91%) patients completed the planned dose of 50–50.4 Gy; in addition, two (2%) patients received 42 Gy, two (2%) patients received 44 Gy, three (3%) patients received 46 Gy, and two (2%) patients received 48 Gy. A total of 90 (93%) patients completed 2 cycles of chemotherapy and the rest 7 (7%) patients underwent 1 cycle of chemotherapy. At the time of analysis, the median period of follow-up was for 60.3 months for the 18 survivors and 15.3 months (range: 2.2–96 months) for all 97 patients. The clinicopathological parameters of the patients are shown in Table 1.

**Table 1:** Characteristics of 97 locally advanced esophageal squamous cell carcinoma patients who received curative concurrent chemoradiotherapy

Characteristics	
Age	56 years old (32–77)
Sex	
Male	96 (99%)
Female	1 (1%)
T status	
1	4 (4%)
2	11 (11%)
3	29 (30%)
4	53 (55%)
N status	
0	4 (4%)
1	27 (28%)
2	39 (40%)
3	27 (28%)
Stage	
II	4 (4%)
IIIA	13 (13%)
IIIB	14 (15%)
IIIC	66 (68%)
Grade	
1	23 (24%)
2	48 (49%)
3	26 (27%)
Location	
Upper	40 (41%)
Middle	34 (35%)
Lower	23 (24%)
Radiotherapy dose	
42 Gy	2 (2%)
44 Gy	2 (2%)
46 Gy	3 (3%)
48 Gy	2 (2%)
50–50.4 Gy	88 (91%)
Cycles of chemotherapy	
1 cycle	7 (7%)
2 cycles	90 (93%)

### Clinical outcomes of ESCC patients receiving curative CCRT

**Fig. 1:**
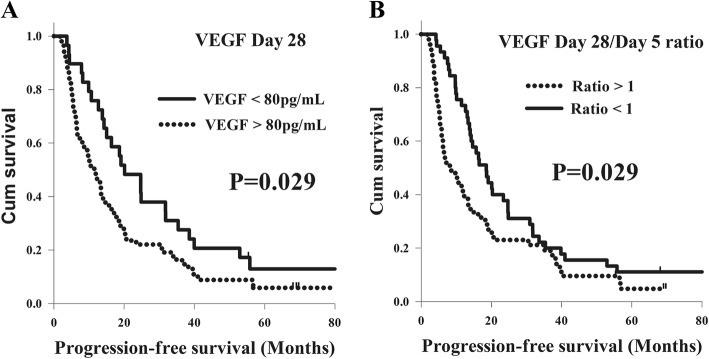
Comparison of progression-free survival curves for 97 esophageal squamous cell carcinoma patients in different VEGF groups. **a** VEGF day 28 value. **b** VEGF day 28/day 5 ratio

With respect to PFS, there were no significant differences in terms of T status, N status, tumor stage, tumor grade, or tumor location in a univariate analysis. Meanwhile, better PFS was found in the total of 64 patients who were less than 60 years old in comparison with the 33 patients who were more than 60 years old (15.6 months versus 11.5 months, *P* = 0.044). Furthermore, the patients with VEGF < 80 pg/mL had superior PFS compared to those with VEGF > 80 pg/mL in the analysis of the day 0, day 5, and day 28 values, but there was only statistical difference in the analysis of day 28 value. The 29 patients who had VEGF < 80 pg/mL had better PFS than the 68 patients with VEGF > 80 pg/mL at day 28 (20.1 months versus 11.5 months, *P* = 0.029, Fig. 1a). In addition, the patients with a day 5/day 0 ratio < 1, a day 28/day 0 ratio < 1, and a day 28/day 5 ratio < 1 had longer PFS than those patients with a ratio > 1, but statistical difference was only mentioned in the analysis of day 28/day 5 ratio. The 45 patients who had a day 28/day 5 ratio < 1 had superior PFS compared to the 52 patients with a day 28/day 5 ratio > 1 (18.5 months versus 8.2 months, P = 0.029, Fig. 1b). Multivariate analysis revealed that young age (*P* = 0.04, hazard ratio: 0.63, 95% confidence interval: 0.40–0.98) was a significant independent factor in better PFS. In addition, a VEGF value < 80 pg/mL at day 28 (*P* = 0.028, hazard ratio: 0.59, 95% confidence interval: 0.37–0.94) and a day 28/day 5 ratio < 1 (*P* = 0.017, hazard ratio: 0.59, 95% confidence interval: 0.39–0.91) represented independent predictive factors of superior PFS, respectively. The analyses results of PFS in the 97 ESCC patients are shown in Table 2.

**Table 2:** Univariate analysis results of progression-free survival in in 97 locally advanced esophageal squamous cell carcinoma patients who received curative concurrent chemoradiotherapy

Characteristics	No. of patients	Median PFS (months)	*P* value
Age			
< 60 years	64 (66%)	15.6	0.044^a^
≥ 60 years	33 (34%)	11.5	
T status			
1 + 2	15 (16%)	19.1	0.35
3 + 4	82 (84%)	11.9	
N status			
0 + 1	32 (33%)	20.3	0.38
2 + 3	65 (67%)	11.9	
Stage			
II + IIIA + IIIB	31 (32%)	16.5	0.93
IIIC	66 (68%)	11.5	
Grade			
1 + 2	71 (73%)	13.3	0.49
3	26 (27%)	14.0	
Location			
Upper	40 (41%)	14.6	0.76
Middle + Lower	57 (59%)	12.3	
VEGF Day 0 (Mean: 176 pg/mL)			
< 80	33 (34%)	14.2	0.20
> 80	64 (66%)	13.3	
VEGF Day 5 (Mean: 163 pg/mL)			
< 80	33 (34%)	18.2	0.39
> 80	64 (66%)	11.9	
VEGF Day 28 (Mean: 195 pg/mL)			
< 80	29 (30%)	20.1	0.029^a^
> 80	68 (70%)	11.5	
VEGF Day 5/Day 0 ratio			
< 1	52 (54%)	15.0	0.46
> 1	45 (46%)	10.2	
VEGF Day 28/Day 0 ratio			
< 1	39 (40%)	16.3	0.55
> 1	58 (60%)	11.5	
VEGF Day 28/Day 5 ratio			
< 1	45 (46%)	18.5	0.029^a^
> 1	52 (54%)	8.2	

*PFS* progression-free survival; *VEGF* vascular endothelial growth factor; *HR* hazard ratio; *CI* confidence interval

^a^Statistically significant

**Fig. 2:**
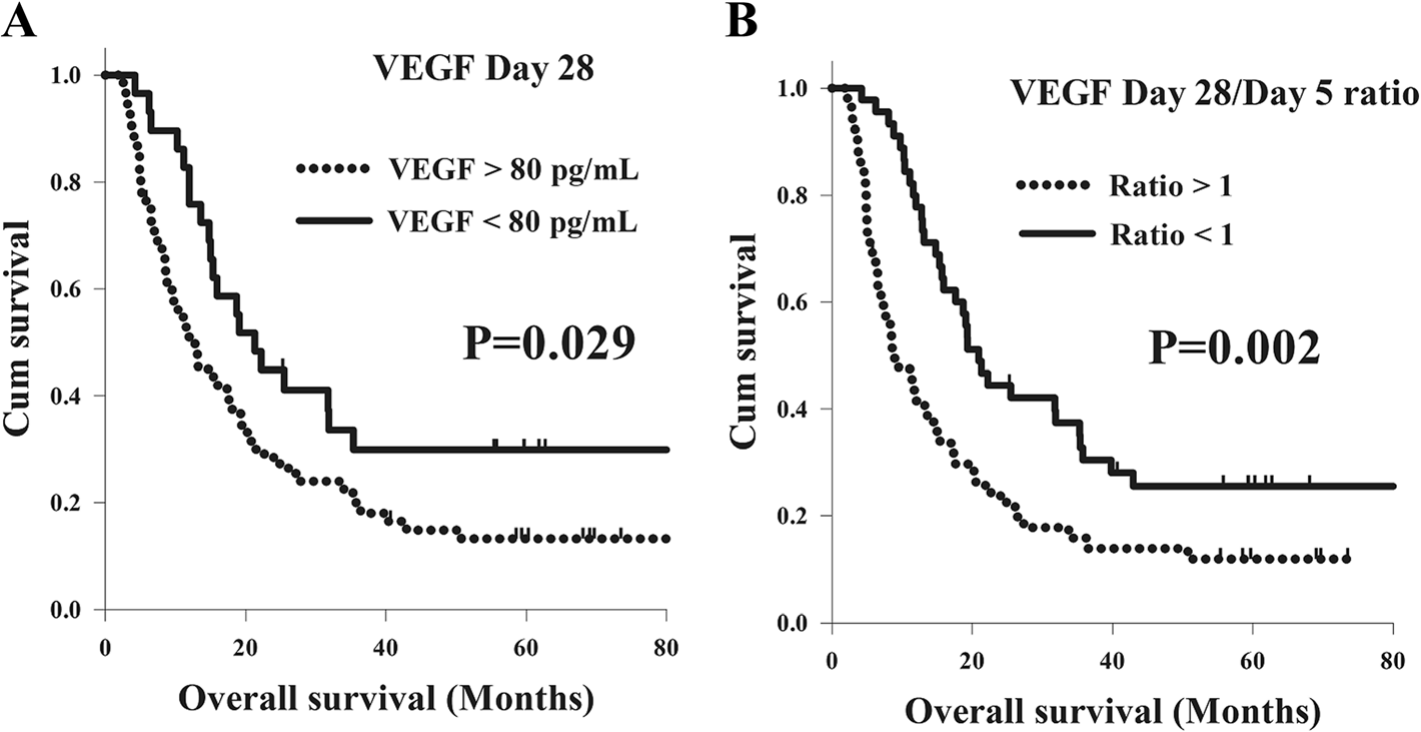
Comparison of overall survival curves for 97 esophageal squamous cell carcinoma patients in different VEGF groups. **a** VEGF day 28 value. **b** VEGF day 28/day 5 ratio

With respect to OS, there were no significant differences in OS in terms of age, T status, tumor stage, tumor grade, or tumor location in a univariate analysis. Meanwhile, better OS was found in the total of 32 patients with N0–1 status in comparison with the 65 patients with N2–3 status (25.5 months versus 12.9 months, *P* = 0.021). The 29 patients who had VEGF < 80 pg/mL had superior OS than the 68 patients with VEGF > 80 pg/mL (21.3 months versus 12.8 months, *P* = 0.029, Fig. 2a). In addition, the patients with a day 5/day 0 ratio < 1, a day 28/day 0 ratio < 1, and a day 28/day 5 ratio < 1 had longer OS than those patients with ratio > 1, but statistical difference was only mentioned in the analysis of day 5/day 0 ratio and day 28/day 5 ratio. The 52 patients with a day 5/day 0 ratio < 1 had longer OS than the 45 patients with a day 5/day 0 ratio > 1 (19.3 months versus 12.0 months, *P* = 0.026). The 45 patients who had a day 28/day 5 ratio < 1 had superior OS compared to the 52 patients with a day 28/day 5 ratio > 1 (20.9 months versus 8.6 months, *P* = 0.002, Fig. 2b). According to a multivariate comparison, N0–1 status (*P* = 0.008, hazard ratio: 0.51, 95% confidence interval: 0.31–0.84) was a significant independent factor in better OS. In addition, a VEGF value < 80 pg/mL at day 28 (*P* = 0.014, hazard ratio: 0.52, 95% confidence interval: 0.31–0.88) and a day 28/day 5 ratio < 1 (*P* = 0.001, hazard ratio: 0.46, 95% confidence interval: 0.29–0.73) represented independent predictive factors of superior OS, respectively. The analyses results of OS in the 97 ESCC patients are shown in Table 3.

**Table 3:** Univariate analysis results of overall survival in 97 locally advanced esophageal squamous cell carcinoma patients who received curative concurrent chemoradiotherapy

Characteristics	No. of patients	Median OS (months)	*P* value
Age			
< 60 years	64 (66%)	17.3	0.09
≥ 60 years	33 (34%)	13.1	
T status			
1 + 2	15 (16%)	19.3	0.30
3 + 4	82 (84%)	13.6	
N status			
0 + 1	32 (33%)	25.5	0.021^a^
2 + 3	65 (67%)	12.9	
Stage			
II + IIIA + IIIB	31 (32%)	19.3	0.47
IIIC	66 (68%)	13.1	
Grade			
1 + 2	71 (73%)	15.6	0.76
3	26 (27%)	13.6	
Location			
Upper	40 (41%)	19.3	0.42
Middle + Lower	57 (59%)	13.2	
VEGF Day 0 (Mean: 176 pg/mL)			
< 80	33 (34%)	15.0	0.52
> 80	64 (66%)	15.6	
VEGF Day 5 (Mean: 163 pg/mL)			
< 80	33 (34%)	22.6	0.06
> 80	64 (66%)	12.0	
VEGF Day 28 (Mean: 195 pg/mL)			
< 80	29 (30%)	21.3	0.029^a^
> 80	68 (70%)	12.8	
VEGF Day 5/Day 0 ratio			
< 1	52 (54%)	19.3	0.026^a^
> 1	45 (46%)	12.0	
VEGF Day 28/Day 0 ratio			
< 1	39 (40%)	12.9	0.43
> 1	58 (60%)	17.6	
VEGF Day 28/Day 5 ratio			
< 1	45 (46%)	20.9	0.002^a^
> 1	52 (54%)	8.6	

*OS* overall survival; *VEGF* vascular endothelial growth factor; *HR* hazard ratio; *CI* confidence interval

^a^Statistically significant

### Comparison between ESCC patients with increased and decreased VEGF

According to the survival analysis of PFS and OS, the VEGF day 28 value and VEGF day 28/day 5 ratio were the only two significant prognostic factors for both PFS and OS. In the analysis of the VEGF day 28 values, the baseline characteristics did not differ significantly between the VEGF < 80 pg/mL and VEGF > 80 pg/mL groups, including in terms of age, N status, tumor stage, tumor grade, and tumor location, with the exception of T status. The VEGF > 80 pg/mL group included a higher percentage of patients with advanced T status than the VEGF < 80 pg/mL group (90% versus 72%, *P* = 0.031). The clinicopathological parameters of these patients are shown in Table 4. Moreover, in the analysis of the VEGF day 28/day 5 ratios, there were no significant difference in the baseline characteristics between the ratio < 1 and ratio > 1 groups, including in terms of age, T status, N status, tumor stage, tumor grade, and tumor location. The clinicopathological parameters of these patients are shown in Table 5.

**Table 4:** Comparison of clinicopathological parameters of 97 locally advanced esophageal squamous cell carcinoma patients who received curative concurrent chemoradiotherapy according to VEGF day 28 level

Characteristics	VEGF < 80 pg/mL group (*N* = 29)	VEGF > 80 pg/mL group (*N* = 68)	*P* value
Age			
< 60 years	21 (72%)	43 (63%)	0.38
≥ 60 years	8 (28%)	25 (37%)	
T status			
1 + 2	8 (28%)	7 (10%)	0.031^a^
3 + 4	21 (72%)	61 (90%)	
N status			
0 + 1	9 (31%)	22 (32%)	0.90
2 + 3	20 (69%)	46 (68%)	
Stage			
II + IIIA + IIIB	11 (38%)	21 (31%)	0.50
IIIC	18 (62%)	47 (69%)	
Grade			
1 + 2	22 (76%)	49 (72%)	0.70
3	7 (24%)	19 (28%)	
Location			
Upper	14 (48%)	26 (38%)	0.36
Middle + Lower	15 (52%)	42 (62%)	

*VEGF* vascular endothelial growth factor

^a^Statistically significant

**Table 5:** Comparison of clinicopathological parameters of 97 locally advanced esophageal squamous cell carcinoma patients who received curative concurrent chemoradiotherapy according to VEGF day 28/day 5 ratio

Characteristics	Day 28/Day5 ratio < 1 group (*N* = 45)	Day 28/Day5 ratio > 1 group (*N* = 52)	*P* value
Age			
< 60 years	29 (64%)	35 (67%)	0.77
≥ 60 years	16 (36%)	17 (33%)	
T status			
1 + 2	8 (18%)	7 (14%)	0.56
3 + 4	37 (82%)	45 (86%)	
N status			
0 + 1	14 (31%)	17 (33%)	0.87
2 + 3	31 (69%)	35 (67%)	
Stage			
II + IIIA + IIIB	17 (38%)	15 (29%)	0.35
IIIC	28 (62%)	37 (71%)	
Grade			
1 + 2	35 (78%)	36 (69%)	0.34
3	10 (22%)	16 (31%)	
Location			
Upper	22 (49%)	18 (35%)	0.15
Middle + Lower	23 (51%)	34 (65%)	

*VEGF* vascular endothelial growth factor

## Discussion

The role of angiogenesis in the tumor growth and progression of cancer has been well documented, and angiogenesis is regulated by a balance between activator and inhibitor molecules, which are produced by both tumor cells, stroma and host cells. As angiogenesis is the central part of tumor growth and disease progression, the proliferation, migration, invasion, and metastasis of tumor cells are associated with angiogenic activity. Therefore, there may be a relationship between the responsiveness to a given anti-cancer treatment, such as chemotherapy or radiotherapy, and angiogenic activity. Several studies have shown that assessments of angiogenic activity may predict treatment responses to chemotherapy or radiotherapy and provide independent prognostic factors, such as tumor size, lymph node involvement, or distant metastasis [18–21]. VEGF is one of the most important biomarker of angiogenesis, and the clinical impact of circulating VEGF has been extensively studied [22–25].

Several studies have investigated the relationship between angiogenesis and tumor responses to treatments such as chemotherapy or radiotherapy in several types of cancer. Dirix et al. reported that serum VEGF levels were higher in cases of progressive disease than in cases of responsive disease in untreated and treated metastatic cancer patients [26]. Another study, reported by Hyodo, demonstrated that low plasma VEGF levels were significant associated with higher responsiveness to chemotherapy and better OS in patients with gastrointestinal cancer [27]. Subsequently, a Japanese study revealed that high serum levels of VEGF were found to be associated with tumor progression, poor treatment response, and poor survival in esophageal cancer patients [28]. For ESCC patients, a Chinese study showed that serum VEGF level was higher in ESCC patients than in health control, and the changes of serum VEGF level before and after treatment may provide prognostic information [29]. In contrast, VEGF was regarded as a negative biomarker in a systematic review; however, these tests have been studied by different methods at different institutions in different populations, so a large prospective study will be needed to confirm the reliability in the future [30]. In theory, measurements of circulating VEGF may be significant in predicting tumor responses to anti-cancer therapies, but there have been some limitations in using such measurements for that purpose so far. For example, the prognostic and predictive significance reported by several studies are not consistent, such that the clinical relevance of serum levels of VEGF among different studies in different cancer types varies extensively. In addition, the serum VEGF levels of cancer patients and healthy people may be overlapped. Therefore, pre-treatment serum VEGF levels do not effectively predict treatment responses, and defining an optimal cutoff value for use in clinical practice is very difficult, contributing to limit the potential of such levels to serve as a clinically valuable biomarker. However, growing evidences have shown that post-treatment changes in circulating VEGF levels are highly related to responses to treatment, such that it may be quite significant to monitor responses to anti-cancer therapies in clinical practice according to post-treatment changes in circulating serum VEGF levels [31–33]. In this study, we found that ESCC patients with lower post-treatment VEGF levels and post-treatment/pre-treatment VEGF ratios < 1 had both superior PFS and OS, and this finding was also compatible with the findings of previous studies.

In the present study, ESCC patients with lower T status had longer PFS and OS than those with advanced T status, but there was no statistical significance to these differences. In addition, the effect of lymph node metastasis is also controversial; N status is a prognostic factor in the analysis of OS, but in this study, there was no significant difference in the analysis results of PFS between the ESCC patients with higher and lower N statuses, although PFS was still longer in the patients with lower N status than those with higher N status. Moreover, patients with VEGF < 80 pg/mL at day 0, day 5, and day 28 had better PFS and OS than those with VEGF > 80 pg/mL, although statistical significance was only mentioned at day 28 value. The same findings were also found in the analysis of post-treatment/pre-treatment VEGF ratios. Patients who had a ratio < 1 at day 5/day 0, day 28/day 0, and day 28/day 5 had superior PFS and OS than those with ratio > 1; however, the difference was only significant for the day 28/day 5 ratios. These results may be related, however, to the small number of patients included in the study.

Our study had several limitations. First, it was a retrospective study of patients treated at a single institution, and the number of enrolled patients was relatively small. Second, there was no information regarding VEGF levels when these patients experienced disease progression, so the correlations, if any, between tumor progression and the sequential changes in VEGF were unclear. Third, because of the limited number of patients, non-bias matching analysis was not available when using the propensity score matching method. Currently, most studies focus on the role of VEGF in monitoring responses and predicting the outcomes of treatment, and measurements of the sequential changes in VEGF may be useful in selecting patients for specific therapies. Nonetheless, to the best of our knowledge, this study constitutes the largest series thus far to investigate the role of VEGF changes in ESCC patients who underwent curative CCRT, and it may thus be useful for understanding the novel mechanism of the investigated disease entity.

## Conclusion

The results of our study suggest that VEGF kinetics is a prognostic factor for locally advanced ESCC patients receiving curative CCRT. For these patients, lower post-treatment VEGF levels and decreasing levels of VEGF during CCRT are significantly associated with better clinical outcomes.

## Abbreviations

^18^F-FDG: 18-fluorodeoxy glucose
CCRT: Curative concurrent chemoradiotherapy
CT: Computed tomography
CTV: Clinical target volume
ESCC: Esophageal squamous cell carcinoma
EUS: Endoscopic ultrasonography
GTV: Gross target volume
IMRT: Intensity-modulated radiotherapy
PET: Positron emission tomography
PTV: Planning target volume
RT: Radiotherapy

## References

[1] National Department of Health, Republic of China (2015). Cancer Registry Annual Report.

[2] Burmeister BH, Smithers BM, Gebski V, Fitzgerald L, Simes RJ, Devitt P, Ackland S, Gotley DC, Joseph D, Millar J (2005). Surgery alone versus chemoradiotherapy followed by surgery for resectable cancer of the oesophagus: a randomised controlled phase III trial. The Lancet Oncology.

[3] Hsu PK, Wu YC, Chou TY, Huang CS, Hsu WH (2010). Comparison of the 6th and 7th editions of the American joint committee on Cancer tumor-node-metastasis staging system in patients with resected esophageal carcinoma. Ann Thorac Surg.

[4] Kelsen DP, Ginsberg R, Pajak TF, Sheahan DG, Gunderson L, Mortimer J, Estes N, Haller DG, Ajani J, Kocha W (1998). Chemotherapy followed by surgery compared with surgery alone for localized esophageal cancer. N Engl J Med.

[5] Kelsen DP, Winter KA, Gunderson LL, Mortimer J, Estes NC, Haller DG, Ajani JA, Kocha W, Minsky BD, Roth JA (2007). Long-term results of RTOG trial 8911 (USA intergroup 113): a random assignment trial comparison of chemotherapy followed by surgery compared with surgery alone for esophageal cancer. J Clin Oncol Off J Am Soc Clin Oncol.

[6] Medical Research Council Oesophageal Cancer Working G (2002). Surgical resection with or without preoperative chemotherapy in oesophageal cancer: a randomised controlled trial. Lancet.

[7] Folkman J, Merler E, Abernathy C, Williams G (1971). Isolation of a tumor factor responsible for angiogenesis. J Exp Med.

[8] Chen X, Zheng Z, Chen L, Zheng H (2017). MAPK, NFkappaB, and VEGF signaling pathways regulate breast cancer liver metastasis. Oncotarget.

[9] Kim KJ, Li B, Winer J, Armanini M, Gillett N, Phillips HS, Ferrara N (1993). Inhibition of vascular endothelial growth factor-induced angiogenesis suppresses tumour growth in vivo. Nature.

[10] Kotowicz B, Fuksiewicz M, Jonska-Gmyrek J, Berezowska A, Radziszewski J, Bidzinski M, Kowalska M (2017). Clinical significance of pretreatment serum levels of VEGF and its receptors, IL- 8, and their prognostic value in type I and II endometrial cancer patients. PLoS One.

[11] Schirosi L, De Summa S, Tommasi S, Paradiso A, Gasparini G, Popescu O, Simone G, Mangia A (2017). VEGF and TWIST1 in a 16-biomarker immunoprofile useful for prognosis of breast cancer patients. Int J Cancer..

[12] Imdahl A, Bognar G, Schulte-Monting J, Schoffel U, Farthmann EH, Ihling C (2002). Predictive factors for response to neoadjuvant therapy in patients with oesophageal cancer. Eur J Cardiothorac Surg.

[13] Shimada H, Hoshino T, Okazumi S, Matsubara H, Funami Y, Nabeya Y, Hayashi H, Takeda A, Shiratori T, Uno T (2002). Expression of angiogenic factors predicts response to chemoradiotherapy and prognosis of oesophageal squamous cell carcinoma. Br J Cancer.

[14] Kozin SV, Boucher Y, Hicklin DJ, Bohlen P, Jain RK, Suit HD (2001). Vascular endothelial growth factor receptor-2-blocking antibody potentiates radiation-induced long-term control of human tumor xenografts. Cancer Res.

[15] Chen YH, Lu HI, Lo CM, Wang YM, Chou SY, Huang CH, Shih LH, Chen SW, Li SH (2018). The clinical impact of supraclavicular lymph node metastasis in patients with locally advanced esophageal squamous cell carcinoma receiving curative concurrent chemoradiotherapy. PLoS One.

[16] Chen YHLH, Wang YM, Lo CM, Chou SY, Huang CH, Shih LH, Chen SW, Li SH (2017). The prognostic significance of celiac lymph node metastasis in patients with locally advanced esophageal squamous cell carcinoma receiving curative concurrent chemoradiotherapy. Oncotarget.

[17] Wang J, Yu JP, Wang JL, Ni XC, Sun ZQ, Sun W, Nie B, Jiang JT, Sun SP, Wu CP (2016). pathologic response and changes of serum VEGF during chemoradiotherapy may predict prognosis in non-surgical patients with esophageal carcinoma. Zhonghua Zhong Liu Za Zhi.

[18] Ellis LM, Takahashi Y, Fenoglio CJ, Cleary KR, Bucana CD, Evans DB (1998). Vessel counts and vascular endothelial growth factor expression in pancreatic adenocarcinoma. Eur J Cancer.

[19] Igarashi M, Dhar DK, Kubota H, Yamamoto A, El-Assal O, Nagasue N (1998). The prognostic significance of microvessel density and thymidine phosphorylase expression in squamous cell carcinoma of the esophagus. Cancer.

[20] Maeda K, Chung YS, Takatsuka S, Ogawa Y, Sawada T, Yamashita Y, Onoda N, Kato Y, Nitta A, Arimoto Y (1995). Tumor angiogenesis as a predictor of recurrence in gastric carcinoma. J Clin Oncol Off J Am Soc Clin Oncol.

[21] Takebayashi Y, Akiyama S, Akiba S, Yamada K, Miyadera K, Sumizawa T, Yamada Y, Murata F, Aikou T (1996). Clinicopathologic and prognostic significance of an angiogenic factor, thymidine phosphorylase, in human colorectal carcinoma. J Natl Cancer Inst.

[22] Aguayo A, Kantarjian HM, Estey EH, Giles FJ, Verstovsek S, Manshouri T, Gidel C, O'Brien S, Keating MJ, Albitar M (2002). Plasma vascular endothelial growth factor levels have prognostic significance in patients with acute myeloid leukemia but not in patients with myelodysplastic syndromes. Cancer.

[23] Jacobsen J, Rasmuson T, Grankvist K, Ljungberg B (2000). Vascular endothelial growth factor as prognostic factor in renal cell carcinoma. J Urol.

[24] Werther K, Christensen IJ, Nielsen HJ, Danish RCCSG (2002). Prognostic impact of matched preoperative plasma and serum VEGF in patients with primary colorectal carcinoma. Br J Cancer.

[25] Yoshikawa T, Tsuburaya A, Kobayashi O, Sairenji M, Motohashi H, Yanoma S, Noguchi Y (2000). Plasma concentrations of VEGF and bFGF in patients with gastric carcinoma. Cancer Lett.

[26] Dirix LY, Vermeulen PB, Pawinski A, Prove A, Benoy I, De Pooter C, Martin M, Van Oosterom AT (1997). Elevated levels of the angiogenic cytokines basic fibroblast growth factor and vascular endothelial growth factor in sera of cancer patients. Br J Cancer.

[27] Hyodo I, Doi T, Endo H, Hosokawa Y, Nishikawa Y, Tanimizu M, Jinno K, Kotani Y (1998). Clinical significance of plasma vascular endothelial growth factor in gastrointestinal cancer. Eur J Cancer.

[28] Shimada H, Takeda A, Nabeya Y, Okazumi SI, Matsubara H, Funami Y, Hayashi H, Gunji Y, Kobayashi S, Suzuki T (2001). Clinical significance of serum vascular endothelial growth factor in esophageal squamous cell carcinoma. Cancer.

[29] Wang XS, Liu MZ, Zhang CQ, Cai L, Cui NJ (2006). effect of concurrent chemoradiotherapy on serum vascular endothelial growth factor in esophageal squamous cell carcinoma patients--a report of 43 cases. Ai Zheng.

[30] Sato Y, Motoyama S, Saito H, Minamiya Y (2016). Novel candidate biomarkers of Chemoradiosensitivity in esophageal squamous cell carcinoma: a systematic review. Eur Surg Res.

[31] Drake MJ, Robson W, Mehta P, Schofield I, Neal DE, Leung HY (2003). An open-label phase II study of low-dose thalidomide in androgen-independent prostate cancer. Br J Cancer.

[32] Stadler WM, Cao D, Vogelzang NJ, Ryan CW, Hoving K, Wright R, Karrison T, Vokes EE (2004). A randomized phase II trial of the antiangiogenic agent SU5416 in hormone-refractory prostate cancer. Clin Cancer Res.

[33] Zangari M, Anaissie E, Stopeck A, Morimoto A, Tan N, Lancet J, Cooper M, Hannah A, Garcia-Manero G, Faderl S (2004). Phase II study of SU5416, a small molecule vascular endothelial growth factor tyrosine kinase receptor inhibitor, in patients with refractory multiple myeloma. Clin can res.

